# Global warming and the possible globalization of vector-borne diseases: a call for increased awareness and action

**DOI:** 10.1186/s41182-016-0039-0

**Published:** 2016-11-24

**Authors:** Emmanuel O. Balogun, Andrew J. Nok, Kiyoshi Kita

**Affiliations:** 1 Department of Biomedical Chemistry, Graduate School of Medicine, The University of Tokyo, 7-3-1 Hongo, Bunkyo-Ku, Tokyo, 113-0033 Japan; 2 Department of Biochemistry, Ahmadu Bello University, Zaria, 2222 Nigeria; 3 African Center of Excellence on Neglected Tropical Diseases and Forensic Biotechnology, Ahmadu Bello University, Zaria, 2222 Nigeria; 4 School of Tropical Medicine and Global Health, Nagasaki University, Nagasaki, 852-8523 Japan

**Keywords:** Global health, Global warming, Vector-borne diseases

## Abstract

Human activities such as burning of fossil fuels play a role in upsetting a previously more balanced and harmonious ecosystem. Climate change—a significant variation in the usual pattern of Earth’s average weather conditions is a product of this ecosystem imbalance, and the rise in the Earth’s average temperature (global warming) is a prominent evidence. There is a correlation between global warming and the ease of transmission of infectious diseases. Therefore, with global health in focus, we herein opine a stepping-up of research activities regarding global warming and infectious diseases globally.

## Background

Evidences on the significant rise in the Earth’s temperature (global warming) are accumulating, and the rise is predicted to continue further. This has been shown to be concomitant with the expansion of the foci of infectious diseases most especially those transmitted by vectors such as arthropods. In order to prevent the worldwide spread of the vectors and the diseases they transmit, we herein advice on the need to promote policies that encourage living conditions that are unfavorable to the breeding of infectious disease vectors in developing countries, and to increase the funding for research leading prevention and management interventions for such diseases globally.

## Main text

Two remarkable consequences of technological advancements are (1) the boost in global economy, and (2) global warming. While the former increases access to goods and services and allows populations to enjoy better standards of living; the latter is deleterious to all living things. Aside from the increased physical disturbances such as flooding of coastal towns partly due to raised water levels from melting of glaciers, other impacts abound. An important consequence of global warming on the ecohealth includes higher prevalence and wider spread of infectious diseases of plant and animals. The major group of human infectious diseases known to be most favored by global warming are the vector-borne diseases (VBDs) [1].

VBDs are infectious diseases whose causative agents require other organisms (such as mosquitoes and flies, bugs, and snails) to deliver them to their hosts (such as humans). Once established in the host, they can produce sickness to varying degrees and may result in death. Populations living in poorer countries are much more burdened, causing immense economic losses, human suffering, and death. These diseases, occurring mainly in the tropics are transmissible and are part of a larger group of communicable diseases known as neglected tropical diseases (NTDs). Given that the vectors do not thrive well at low temperatures, temperate regions were protected from the transmission of most VBDs, localizing them to the tropics. Unfortunately, this *transmission barrier* is gradually failing due to global warming. Currently, VBDs are spreading and maintaining transmission foci in regions where they previously did not occur. For example, Europe is presently experiencing a significant surge in new cases of various VBDs [2].

The health impact of global warming is enormous. It has been estimated that a rise in global temperatures by 2 to 3 °C will increase the population risk for malaria by 3 to 5%, meaning that millions of additional people will experience malaria infection each year [3]. This phenomenon persists because the insect vectors tend to thrive better at higher temperatures. This is further amplified by global warming, which will lead to climate-induced shifts in vectors movements, thereby affecting disease dynamics [4, 5] i.e., resulting in the emergence and re-emergence of VBDs. A number of disease vectors are already moving toward higher altitudes that were previously assumed to be uninhabitable for them because of the lower ambient temperature. For instance, the emergence of trypanosomiasis in Jos Plateau, Nigeria has recently been reported [6]. The Plateau, located in Northern Nigeria, is situated at an altitude of about 1280 m and was previously free of tsetse flies and the trypanosomiasis disease that they transmit [7]. Findings from recent surveys show over 20% prevalence of bovine trypanosomiasis across the Plateau [6]. Similarly, there is now an expansion in the habitat range of the Asian tiger mosquito across Europe, which is attributed to the recent outbreaks of Chikungunya in the subregion [8, 9]. Furthermore, it is alarming that the mosquito-borne flavivirus responsible for the West Nile fever first discovered in East Africa in the mid-1930s and neglected (probably for its vector’s localization to the continent at the time), spread, and got introduced to the United States by 1999, and to Argentina, Canada, Romania, and Russia by 2005 [10]. In the light of vector spread and disease emergence/re-emergence, there is urgent need for enhanced vector control and preparedness for rapid and effective disease management strategies.

These examples clearly demonstrate the potential of global warming to contribute towards the eventual globalization of VBDs. We hold the opinion that majority of the VBDs have the potential to spread even more widely as the Earth’s temperature rises. A recent IPCC report further re-iterates that the global temperature may eventually rise 2 °C by the end of this century [11]. Such rise in temperature may be favorable for the spread and globalization of large numbers of VBDs. With the current lack of vaccine and satisfactory chemotherapy for most VBDs, the impacts on global health and the global economy will be enormous and for now, unquantifiable. Put together, these necessitate the stepping-up of concerted efforts towards addressing the global warming-induced global health concern—globalization of VBDs.

In an effort to stem this spread, the UNICEF/UNDP/World Bank/WHO Special Programme for Research and Training in Tropical Diseases (TDR) in partnership with International Development Research Centre (IDRC), Canada, and other donors have pioneered the funding of trans-disciplinary studies on VBDs in the context of socio-ecological variabilities [12]. This joint partnership aims to contribute towards understanding how to be prepared to face this new dimension of global challenges and threats of VBDs. This will undoubtedly help in the sustainable reduction of population health vulnerabilities and increased resilience against risks to VBDs under climate change conditions.

Noteworthy, the increased interconnectedness of the world has produced what is now referred to, as a *global village* i.e., the world has become a single community. Therefore, the spread of diseases or/and their vectors will be much faster than pre-technology era.

## Conclusion

We call on all global health stakeholders and policy-makers to form coordinated efforts that will help in cushioning the impacts of global warming on the global risks of VBDs. Such efforts must involve the national governments of VBD-endemic countries especially in the underdeveloped and developing countries. The governments should be urged to show responsibility to the people by ensuring improvement in sanitary conditions and facilitating access to living conditions that are unfavorable to the breeding of disease vectors. Furthermore, global intervention campaigns for the eradication and eventual elimination of VBDs (such as malaria, trypanosomiases, leishmaniases, dengue fever, yellow fever) should be sustained. These interventions should include research and increased funding towards the discovery and development of drugs, vaccines, or/and anti-vector agents.

## Abbreviations

IPCC: Intergovernmental panel on climate change
VBDs: Vector-borne diseases
